# Association between diabetes mellitus with metabolic syndrome and diabetic microangiopathy

**DOI:** 10.3892/etm.2014.1992

**Published:** 2014-09-29

**Authors:** XIAOYAN ZHANG, XIAOLI CUI, FENGHUA LI, SHUO WANG, XINYU LIU, LICHAO HUI, NA SONG, NANNAN LI

**Affiliations:** Department of Endocrinology, First Affiliated Hospital, Liaoning Medical College, Jinzhou, Liaoning 121001, P.R. China

**Keywords:** diabetes, diabetic microangiopathy, metabolic syndrome

## Abstract

The aim of this study was to investigate the association between diabetes mellitus (DM), mainly type II, with metabolic syndrome (MS) and diabetic nephropathy (DN)/diabetic retinopathy (DR). Based on the analysis of the prevalence of MS, patients with DM were divided into MS and non-MS groups according to the presence or absence of MS. The correlation between DN, DR and certain factors, including gender, age, disease duration and the presence or absence of a family history of MS, were analyzed. The prevalence of MS among the patients with DM was 62.50%. The prevalence of DN was 55.33% in the MS group and that of DR was 26.00%. DN was positively correlated with age, gender, blood pressure, triglyceride (TG), low-density lipoprotein cholesterol (LDL-C) and blood uric acid. DR was positively correlated with traceable disease duration and LDL-C. In conclusion, DM occurred more frequently in concurrence with MS than without MS, and the prevalence of DN/DR in the MS group was higher than that in the non-MS group. Age, gender, blood pressure, TG, LDL-C and blood uric acid were risk factors for DN and the traceable disease duration and LDL-C were risk factors for DR.

## Introduction

At present, diabetes mellitus (DM) is a serious issue in China, and complications associated with the disease are a serious threat to human health. Diabetic nephropathy (DN) and diabetic retinopathy (DR) are the main microvascular complications, causing kidney failure and blindness, respectively. Metabolic syndrome (MS) is a group of metabolic disorders centered around insulin resistance (IR), with basic features including abnormal glucose metabolism, central obesity, lipid disorders and hypertension. The characteristics of MS vary among different ethnic groups on a global scale and among different provinces within the same country. However, the overall prevalence of MS has increased.

The disease causes damage to the vital organs of the body. Although numerous factors lead to atherosclerosis and clinical cardiovascular events, the occurrence of MS is of particular importance; this has been confirmed in a number of studies described below (1). The National Cholesterol Education Program Adult Treatment Panel Third Guide (NCEP-ATP III) reported that the main clinical outcomes of MS are cardiovascular disease (CVD) and the aggravation of the occurrence and development of DN, DR and other microvascular diseases. Gluckman *et al* (2) believe that insulin resistance in the metabolic reaction develops in adults due to a poor prenatal development environment (such as malnutrition). The study thus speculated that growth in the adaptation to a bad developmental environment in the period of developmental plasticity can be generated by a permanent poor development environment, and that these genes could be susceptibility genes to diabetes or other metabolic diseases, thereby increasing the risk for diabetes in adults. Additionally, the prevalence of MS is expected to increase in China due to its high ageing population, and it is believed that it may bring heavy economic burden to families and the society, therefore, the prevention and treatment of MS is something that is required to be investigated (3). However, few conclusive studies exist on the association between DM with MS and diabetic microangiopathy. Thus, the associations between DM with MS and DN/DR were further explored in the present study by analyzing the hospital data from 240 patients recruited for the study in Jinzhou, China.

## Patients and methods

### Study subjects

Data from 240 patients with diabetes, hospitalized in the Department of Endocrinology of the First Affiliated Hospital of Liaoning Medical College (Jinzhou, China), were collected between March and November, 2012. The study population comprised 135 males and 105 females, aged 14–91 years, with a mean age of 55.6±11.1 years. This study was conducted in accordance with the Declaration of Helsinki, and with approval from the Ethics Committee of Liaoning Medical College. Written informed consent was obtained from all participants.

### Inclusion criteria

#### DM

The inclusion criteria for DM were designed in accordance with the 1999 World Health Organization (WHO) diagnostic criteria for diabetes (4). Patients who exhibited the following characteristics were included: i) Typical symptoms of diabetes (polyuria, polydipsia and unexplained weight loss); ii) random blood glucose levels ≥11.1 mmol/l; iii) fasting plasma glucose levels ≥7.0 mmol/l or 2 h post-challenge glucose levels (hPG) in an oral glucose tolerance test ≥11.1 mmol/l. Any atypical symptoms were confirmed on a different day.

#### MS

The inclusion criteria for MS were designed in accordance with the diagnostic criteria for MS proposed by the Chinese Medical Association Diabetes Society (CDS) in 2004 (5): i) Being overweight and/or obese (body mass index ≥25 kg/m^2^); ii) hyperglycemia [fasting plasma glucose levels ≥6.1 mmol/l (110 mg/dl) and/or 2 hPG ≥7.8 mmol/l (140 mg/dl) and/or patients have been diagnosed as diabetic and treated]; iii) hypertension [blood pressure (BP) ≥140/90 mmHg and/or patients have been diagnosed as hypertensive and treated]; iv) dyslipidemia [fasting triglyceride (TG) levels ≥1.7 mmol/l (150 mg/dl) and/or fasting high-density lipoprotein cholesterol (HDL-C) levels <0.9 mmol/l (35 mg/dl) (male) or <1.0 mmol/l (39 mg/dl) (female)]. Three or all of the four standards being fulfilled led to a diagnosis of MS.

#### DN

According to the diagnostic standards of DN in the Mogensen DN diagnostic criteria (6), DN was divided into five phases: Phase I, a high glomerular filtration period characterized by renal hypertrophy, increasing glomerular filtration rate (GFR) and normal urinary albumin excretion rate (UAER); Phase II, a normal albuminuria period with no obvious clinical manifestations, normal UAER (<20 μg/min) in a resting state and slightly increased UAER in the stress state, and normal or slightly elevated GFR; Phase III, persistent microalbuminuria (MAU) with clinical features such as UAER of 20–200 μg/min, urinary albumin quantification of 30–300 mg in 24 h, negative for urine protein in a routine urine test and normal GFR; Phase IV, a clinical proteinuria period with persistent positive results for urine protein, 24 h urine protein >0.5 g, UAER >200 μg/min and a gradual decrease in GFR; Phase V, end-stage, renal failure with further decline in GFR, GFR <15 ml/min or dialysis, often with heavy proteinuria.

#### DR

The DR diagnostic criteria used were based on the diagnostic criteria developed by the Third Sector National Eye Conference in 1985; the staging of DR is shown in Table I.

### Grouping

The subjects were initially divided into two groups according to gender (135 males and 105 females), and the prevalence of MS, DN and DR in the two groups was calculated. Patients were then divided into the MS and non-MS groups based on whether they suffered from MS or not. The prevalence of DR and DN in the two groups was calculated, as well as the prevalence of DN and DR in the two different gender groups.

### Detection index

Information such as age, gender and traceable duration of diabetes of all the subjects was collected following admission. The BP, height and weight of the subjects were measured by the uniformly trained personnel. Fasting venous plasma glucose was measured by the hexokinase method and the enzyme method was utilized to measure fasting TG and total cholesterol (TC). Fasting HDL-C and low-density lipoprotein cholesterol (LDL-C) were measured using the enzyme-modified method and measurements of glycosylated hemoglobin A1c (HbA1c) were obtained using high-performance liquid chromatography. Enzymatic kinetics, electrochemiluminescence and immunonephelometry were used to obtain measurements of serum uric acid, serum connecting peptide and urinary albumin, respectively, and 24 h urinary protein was measured using chromometry. Ophthalmoscopy was performed on selected patients by the same ophthalmologist in the First Affiliated Hospital of Liaoning Medical College, and fundus angiography was performed when necessary.

### Statistical analysis

All data were processed using the SPSS 17.0 statistical software package (SPSS, Inc., Chicago, IL, USA). Age is expressed as the mean ± standard deviation. Comparisons among the prevalence of MS, DN and DR were performed by the χ^2^ test with independent samples. The associations between MS and DN/DR are expressed by non-conditional logistic regression analysis. P<0.05 was considered to be statistically significant.

## Results

### Prevalence of MS in different gender groups

The prevalence of MS among the diabetic patients was 62.50% (150 cases), and the prevalence of MS among the males and females was 66.67% (90 cases) and 57.14% (60 cases), respectively. The difference in prevalence between the two gender groups was not statistically significant (χ^2^=2.286, P>0.10).

### Prevalence of DN or DR in different gender groups

The prevalence for DN among the male and female patients were 53.33% (72 cases) and 40.00% (42 cases), respectively; the difference in prevalence between the two groups was statistically significant (χ^2^=4.211, P<0.05). The prevalence for DR among the male and female patients were 22.22% (30 cases) and 23.81% (25 cases); the difference in prevalence between the two groups was not statistically significant (χ^2^=0.084, P>0.05).

### Prevalence of DN or DR in the MS and non-MS groups

The prevalence of DN in the MS group was 55.33% (83 cases), which was significantly higher than that in the non-MS group (34.44%, 31 cases). The difference in prevalence between the two groups was statistically significant (χ^2^=9.842, P<0.005). The prevalence of DR was 26.00% (39 cases) in the MS group and 17.78% (16 cases) in the non-MS group; the difference in prevalence between the two groups was not statistically significant (χ^2^=2.153, P>0.05).

### Prevalence of DN among the different genders in the MS and non-MS groups

The prevalence of DN among the males and females in the MS group was 58.89% (53 cases) and 50.00% (30 cases), respectively; the difference in prevalence between the two groups was not statistically significant (χ^2^=1.151, P>0.05). The prevalence of DN among the males and females in the non-MS group was 42.22% (19 cases) and 26.67% (12 cases), respectively; this difference was not statistically significant (χ^2^=2.411, P>0.05).

### Prevalence of DR among the different genders in the MS and non-MS groups

The prevalence of DR among the males and females in the MS group was 23.33% (21 cases) and 30.00% (18 cases), respectively; the difference in prevalence between the two groups was not statistically significant (χ^2^=0.832, P>0.05). The prevalence of DR among the males and females in the non-MS group was 20.00% (9 cases) and 5.56% (7 cases), respectively; this difference was not statistically significant (χ^2^=0.304, P>0.05).

### Univariate analysis

Taking the presence or absence of DN or DR as the dependent variable and gender, age, traceable disease duration, systolic BP (SBP), diastolic BP (DBP), TG, HDL-C, HbA1c or blood uric acid as independent variables, univariate logistic regression analysis was performed. DN was positively correlated with age, gender, SBP, DBP, TG, LDL-C and blood serum uric acid (P<0.05) and was negatively correlated with HDL-C (P<0.05) (Table II). DR was positively correlated with traceable duration and LDL-C (P<0.05; Table III).

### Multivariate analysis

Taking the presence or absence of DN or DR as the dependent variable and gender, age, traceable disease duration, SBP, DBP, TG, HDL-C, HbA1c or blood uric acid as independent variables, multivariate logistic regression analysis was performed. Age, uric acid and MS were risk factors for DN (Table IV), and traceable disease duration was a risk factor for DR (Table V).

## Discussion

According to the available information, the prevalence of MS has increased significantly throughout the world. Furthermore, it is expected that the prevalence will increase exponentially in all countries in the next two centuries. MS is affected by numerous factors; as a result, the common features vary in different regions, as well as the used diagnostic criteria. The prevalence of MS in different regions also varies. In the Third National Health and Nutrition Examination Survey (NHANES III) in the USA, the NHANES III criteria were used as the diagnostic criteria for MS. The survey revealed that, in the USA, the prevalence of MS was 23.9% in adults >20 years old, with an approximately equal prevalence in the two genders (7). The prevalence of MS was significantly higher in adults >50 years old (~44%) and in obese subjects (59.6%). In a study in the Middle East, International Diabetes Federation (IDF) and NCEP-ATPIII diagnostic criteria were used to study the prevalence of MS in diabetic and non-diabetic patients. According to the IDF and NCEP-ATPIII criteria, the prevalence of MS in non-diabetic patients was 29.0 and 31.5%, respectively, while the prevalence of MS in diabetic patients was 63.4 and 64.2%, respectively (8). NCEP-ATPIII diagnostic criteria were also used in a study in Canada, with the results showing that the total prevalence of MS was 19.1%. The prevalence among female subjects was 20.5%, which was higher than that among male subjects (17.8%), but this difference was not significant (9). Jia *et al* (10) in China performed an MS survey with Chinese subjects aged 20–74 years in two communities in Shanghai. When the WHO criteria were used to define the standards, the prevalence of MS was 17.14%, while the prevalence was 10.95% when the NCEP-ATPIII diagnostic criteria were used. A prevalence survey of MS among the elderly in five cities, including Chongqing, Jinan and Tianjin, was performed by Li *et al* (11), according to the diagnostic criteria for MS developed by the Joint Commission on Chinese Adult Dyslipidemia in 2007 and based on the recommendations of the CDS in 2004. The results showed that the crude prevalence of MS among the study population was 25.5%, including rates of 23.2% for male and 26.9% for female subjects. In the present study, the hospitalization data from 240 patients with diabetes in Jinzhou were analyzed. The results showed that the prevalence of MS was 62.6%, higher than that of the general population and similar to the prevalence of diabetes in the Middle East. The prevalence was 66.67 and 57.14% for the male and female patients, respectively. With an increasingly aging society in China, the prevalence of MS is expected to further increase, which is likely to bring a heavy economic burden to families and society. Therefore, the prevention and treatment of MS must be prioritized (12). An epidemiological survey conducted in the USA (13) revealed that the risk of cardiovascular and total mortality associated with high SBP in patients with impaired fasting glycemia was significantly increased with the prevalence of MS. The study also revealed that the occurrence of myocardial infarction and stroke can be predicted by the presence of MS in the elderly population. A similar conclusion was also reached in a prospective study in Japan concerning the association between MS and CVD (14). In addition, it was revealed that MS may lead to loss of cognitive function in the elderly.

The number of diabetic patients worldwide has exhibited a more significant growth than that occurring previously, according to a recent study (15). The prevalence of diabetes has also increased significantly. The epidemiologic survey of diabetes in the 14 countries or provinces organized by the CDS during 2007 and 2008 showed that the prevalence of DM in China was 9.7% in adults >20 years old. The total prevalence was ~92.4 million, which was ranked the first in the world. With the increase in the prevalence of DM, in addition to the increases in efforts to control DM, increased awareness of DM and the prolonged survival time of patients with DM, the prevalence of chronic complications associated with DM has also significantly increased. Epidemiological data revealed that the DR prevalence in patients with type 2 diabetes (T2DM) was 25% on a global scale. Chronic complications of diabetes may be associated with a number of other factors, but there is a lack of well-designed, large-scale epidemiological data in China at present. The available information is limited, exists within a small range and is significantly different among regions. A study by Li *et al* (16) showed that the prevalence of DR was 51.3% in Chinese diabetic patients, and the prevalence increased alongside increases in the number of patients with DM and prolonged survival time. A clinical study by Riqiu Chen *et al* (17) showed that the prevalence of DN and DR was 36.47 and 28.86%, respectively. In the present study, the prevalence of DN was 47.5%. The prevalence of DN among the male subjects (53.33%) was significantly higher than that among the females (40.00%). The prevalence of DR was 22.92% overall, 22.22% for males and 23.81% for females. The majority of the factors comprising MS are risk factors for CVDs. A previous study (18) also identified MS to be an independent risk factor for CVDs. It has been indicated that MS may be closely associated with diabetic microangiopathy, and in the study by Isomaa *et al* (19), the results of the prevalence comparisons of MAU and proteinuria in patients with T2DM showed that the prevalence of T2DM with MS was higher than that of T2DM without MS. In the present study, the CDS diagnostic criteria for MS were used to analyze the clinical data of 240 diabetic patients in Jinzhou. The prevalence of DN was 55.33% in the MS group and 34.44% in the non-MS group, indicating a significant difference. The prevalence of DR in the MS group was 26.00%, which was higher than the prevalence in the non-MS group (17.78%); however, no significant difference was indicated. The prevalence comparisons of DN and DR between different genders in the MS and non-MS groups showed similar results.

A number of studies, including the United Kingdom Prospective Diabetes Study and the Diabetes Control and Complications Trial, have shown that the prevalence of microvascular complications associated with diabetes increases significantly with increases in HbA1c and BP (20). The clinical study of Riqiu Chen *et al* (17) also showed that DN was associated with a number of factors, including age, gender, SBP, TG and TC. DR was positively correlated with age, disease duration and other factors. The results in the present study showed that DN was positively correlated with age, SBP, DBP and serum uric acid, while DR was positively correlated with the duration of diabetes. Epidemiological data, qualitative experiments and clinical studies have confirmed that the pathogenic basis underlying MS is IR and obesity, and it has been suggested that IR is more important than obesity in the pathogenesis of MS (21–23). Lipid metabolism may also be involved in the development of DN. Obesity can cause decreases in afferent arteriole resistance by changing the reabsorption of salt by renal tubules, thereby increasing the GFR; this can lead to DN caused by kidney damage in the long-term future (24). The results in the present study showed that DN was positively correlated with TG and LDL-C and negatively correlated with HDL-C. Thus, reducing MAU by reducing weight and regulating blood fat metabolism may delay the development of DN. The present study also revealed that DR was positively correlated with LDL-C. Diabetic microangiopathy shows certain organ-specific features, which mainly occur in the kidney and retina, but the pathology of the other organs, including the brain and lungs, is not evident. Therefore, further study of the organ-specific mechanisms is a novel perspective for finding targets to prevent and treat diabetic microangiopathy.

Domestic and international data have shown that MS is an important and independent risk factor for DN (25–28). It has been shown that DR is positively correlated with TG and LDL-C and negatively correlated with HDL-C. A previous study also showed that DR had an association with numerous factors, including high BP, lipid disorders and high blood sugar (29). Whether MS was an independent factor required further study. Isomaa *et al* (19) found that MS was an independent risk factor for DN, while DR had no significant correlation with MS. In the present study, the associations between MS and DN/DR were consistent with the results of the study by Isomaa *et al* (19).

At present, treatment for MS is mainly initiated to avoid the adverse consequences of the disease, i.e. to prevent the development of clinical CVD and T2DM. Therefore, the treatment of MS was actively based on DM, CVD and the diseases associated with MS (30–33). The basic principles underlying the treatment of MS have reached a consensus, with emphasis on lifestyle intervention, followed by drug treatment. Based on the disease pathogenesis, the prevention and treatment of diabetic microangiopathy have been investigated, and the use of certain pathway inhibitors and Traditional Chinese Medicines has been explored (34). Current strategies include the use of aldose reductase inhibitors, such as tolrestat, and the development of protein kinase C-B subtype-specific inhibitors, such as ruboxistaurin, and inhibitors of diabetic microangiopathy glycation end products (35) and fructose 6-phosphate acyltransferase (a key enzyme of the hexosamine pathway). In recent years, with the increased prevalence of microvascular diseases associated with diabetes, the results of the prevention and treatment of these diseases by Chinese medicines, including puerarin, *Ginkgo biloba* and curcumin, have been noteworthy. Studies investigating these medicines suggested that they led to a certain degree of improvement in the condition of patients with diabetic microangiopathy.

The results of the present study demonstrated that the prevalence of MS among diabetic patients was high. The prevalence of microvascular complications in the MS group was significantly higher compared to that in the non-MS group. In order to prevent the occurrence and development of diabetic microvascular complications, the active and effective control of the prevalence of MS is necessary.

## Figures and Tables

**Table I:** DR staging criteria developed by the Third Sector National Eye Conference in 1985.

Typing	Staging	Retinopathy
Simple DR, also known as background DR	I: Microaneurysms, punctate and hemorrhage	(+): Less, easy to be counted. (++): More, difficult to be counted
	II: Hard exudates and/or bleeding	(+): Less, easy to be counted. (++): More, difficult to be counted
	III: Cotton wool turbidity (soft exudates) and/or bleeding	(+): Less, easy to be counted. (++): More, difficult to be counted
Proliferative DR	IV	Retinal neovascularization or vitreous hemorrhage
	V	Retinal neovascularization and fibrous proliferation
	VI	Retinal neovascularization and fibrous proliferation, retinal detachment

DR, diabetic retinopathy.

**Table II:** Univariate logistic regression analysis between diabetic nephropathy and single factors.

Variable	β	SE	Wald (χ^2^)	P-value	OR	95% CI OR
Age	0.023	0.009	6.152	0.013	1.023	1.005–1.041
Gender	0.033	0.062	4.211	0.041	0.849	0.388–1.085
Traceable disease duration	0.035	0.021	2.664	0.130	1.035	0.993–1.079
Family history	0.064	0.186	0.852	0.356	0.768	0.438–1.345
SBP	0.037	0.008	22.245	<0.001	1.038	1.022–1.054
DBP	0.041	0.013	10.158	0.001	1.042	1.016–1.068
BMI	0.019	0.035	0.280	0.597	1.019	0.951–1.091
TG	0.188	0.081	5.456	0.019	1.207	1.031–1.413
TC	0.005	0.008	0.395	0.530	0.995	0.978–1.011
LDL-C	0.360	0.157	5.276	0.022	1.433	1.054–1.949
HDL-C	−0.018	0.028	0.404	0.025	0.983	0.931–1.037
HbA1c	0.023	0.035	0.435	0.510	0.977	0.911–1.047
Blood uric acid	0.003	0.001	5.178	0.023	1.003	1.000–1.006
C peptide	0.040	0.063	0.401	0.526	1.041	0.919–1.179
Blood sugar	0.010	0.012	0.650	0.420	1.010	0.986–1.034

SE, standard error; OR, odds ratio; CI, confidence interval; SBP, systolic blood pressure; DBP, diastolic blood pressure; BMI, body mass index; TG, triglyceride; TC, total cholesterol; LDL-C, low-density lipoprotein cholesterol; HDL-C, high-density lipoprotein cholesterol; HbA1c, glycosylated hemoglobin A1c; C peptide, connecting peptide.

**Table III:** Univariate logistic regression analysis between diabetic retinopathy and single factors.

Variable	β	SE	Wald (χ^2^)	P-value	OR	95% CI OR
Age	0.017	0.011	2.578	0.108	1.018	0.996–1.040
Gender	0.163	0.308	0.279	0.579	1.177	0.143–2.153
Traceable disease duration	0.128	0.026	24.223	<0.001	1.136	1.080–1.196
Family history	0.028	0.172	2.843	0.092	0.534	0.257–1.107
SBP	0.010	0.008	1.625	0.202	1.010	0.995–1.025
DBP	0.006	0.014	0.194	0.660	1.006	0.979–1.034
BMI	0.030	0.042	0.514	0.474	1.030	0.950–1.118
TG	0.035	0.092	0.413	0.705	0.966	0.807–1.156
TC	0.203	0.113	3.225	0.073	1.225	0.982–1.528
LDL-C	0.386	0.181	4.562	0.033	1.471	1.032–2.097
HDL-C	−0.021	0.058	0.127	0.721	0.979	0.874–1.098
HbA1c	0.040	0.045	0.182	0.376	0.961	0.880–1.049
Blood uric acid	0.001	0.101	0.611	0.434	1.001	0.998–1.004
C peptide	0.023	0.073	0.095	0.757	1.023	0.886–1.181
Blood sugar	0.043	0.030	2.043	0.153	0.958	0.903–1.016

SE, standard error; OR, odds ratio; CI, confidence interval; SBP, systolic blood pressure; DBP, diastolic blood pressure; BMI, body mass index; TG, triglyceride; TC, total cholesterol; LDL-C, low-density lipoprotein cholesterol; HDL-C, high-density lipoprotein cholesterol; HbA1c, glycosylated hemoglobin A1c; C peptide, connecting peptide.

**Table IV:** Multivariate logistic regression analysis between diabetic nephropathy and multiple factors.

Variable	β	SE	Wald (χ^2^)	P-value	OR	95% CI OR
Age	0.028	0.011	6.856	0.009	1.029	1.007–1.051
Gender	0.380	0.292	2.102	0.207	0.784	0.386–1.211
Traceable disease duration	0.016	0.024	0.437	0.509	1.016	0.969–1.066
Blood uric acid	0.003	0.001	4.463	0.035	1.003	1.000–1.006
MS	0.847	0.294	8.282	0.004	2.333	1.310–4.156

SE, standard error; OR, odds ratio; CI, confidence interval; MS, metabolic syndrome.

**Table V:** Multivariate logistic regression analysis between diabetic retinopathy and multiple factors.

Variable	β	SE	Wald (χ^2^)	P-value	OR	95% CI OR
Age	0.005	0.013	0.120	0.729	0.995	0.970–1.021
Gender	0.144	0.347	0.172	0.679	1.155	0.585–2.281
Traceable disease duration	0.134	0.029	21.479	<0.001	1.143	1.080–1.210
Blood uric acid	0.002	0.002	1.498	0.221	1.002	0.999–1.005
MS	0.360	0.361	0.996	0.318	1.434	0.707–2.909

SE, standard error; OR, odds ratio; CI, confidence interval; MS, metabolic syndrome.
